# Supplementary figures and images for: Sex-specific effects of polygenic risk for schizophrenia on lifespan cognitive functioning in healthy individuals
Source: Transl Psychiatry. 2021 Oct 11;11:520. doi: 10.1038/s41398-021-01649-4 (PMC8505489; doi:10.1038/s41398-021-01649-4)

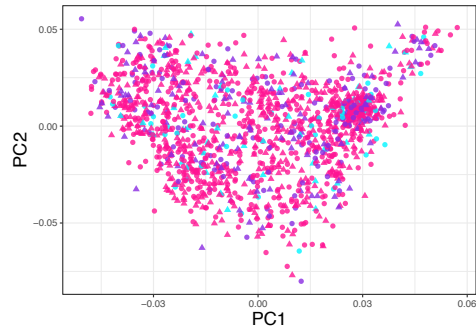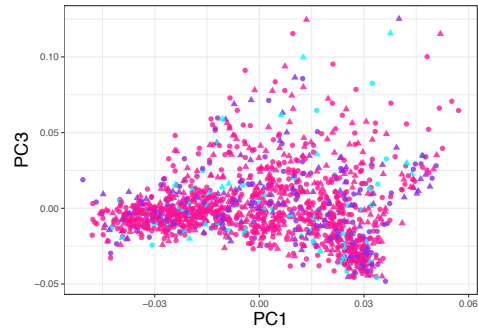

Dataset

- batch 1
- batch 1 and 2
- batch 2

Sex

- female
- male

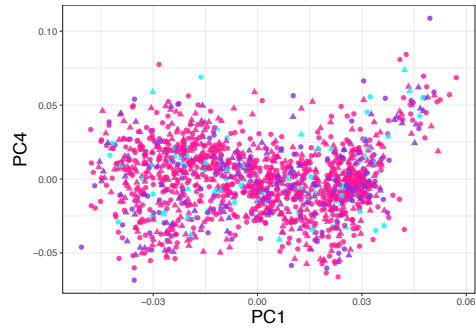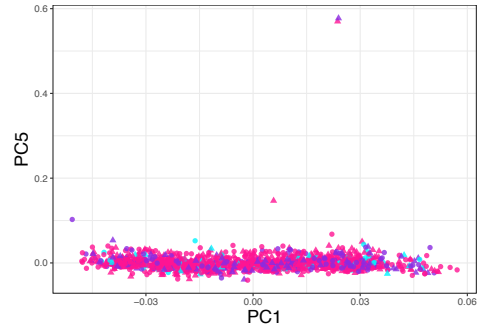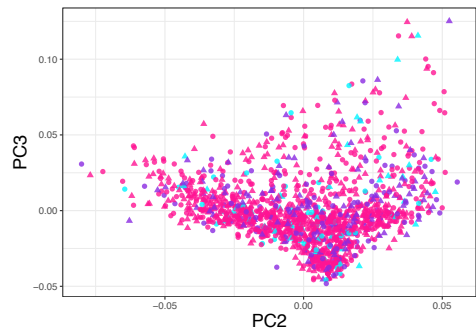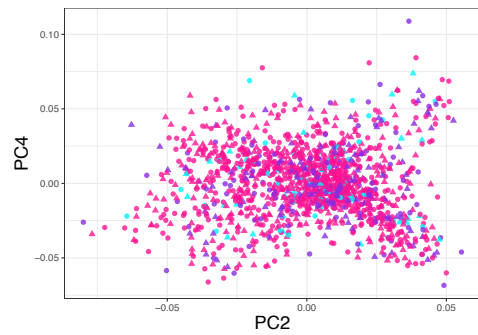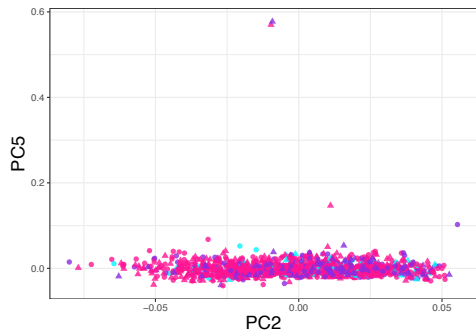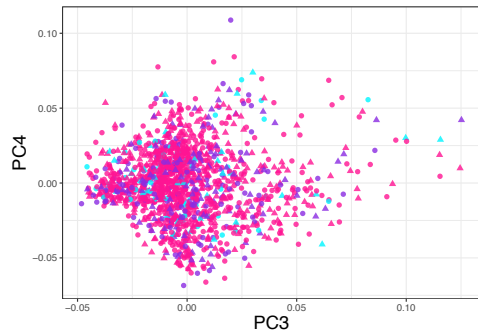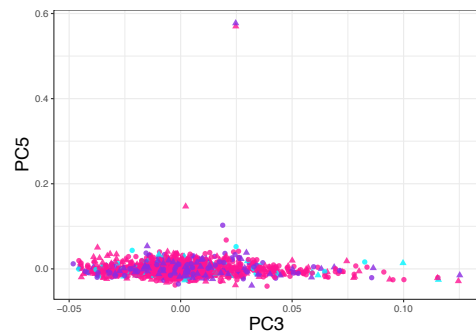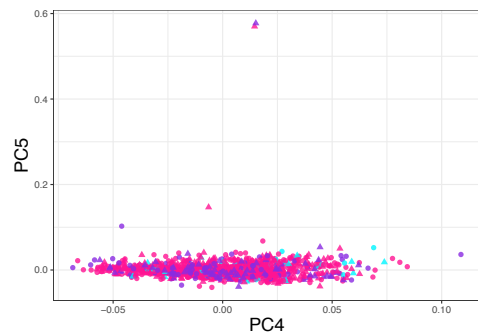

Supplement: Supplementary file 2 — Figure S1 [file 41398_2021_1649_MOESM2_ESM.pdf]

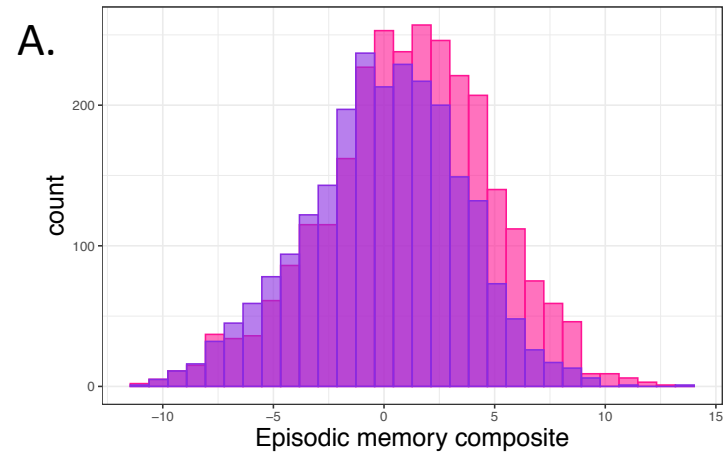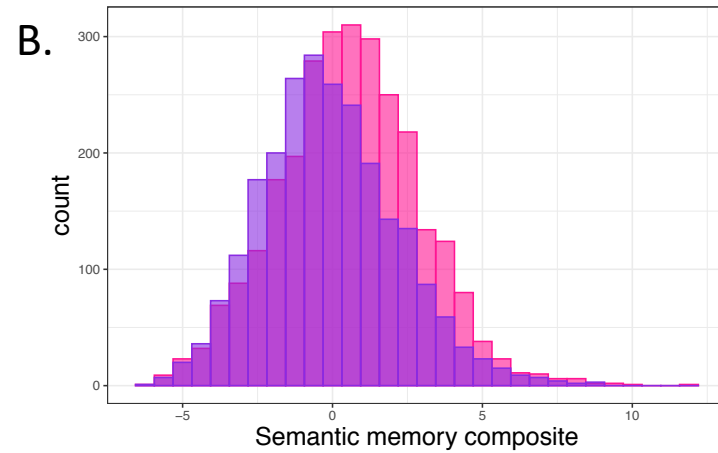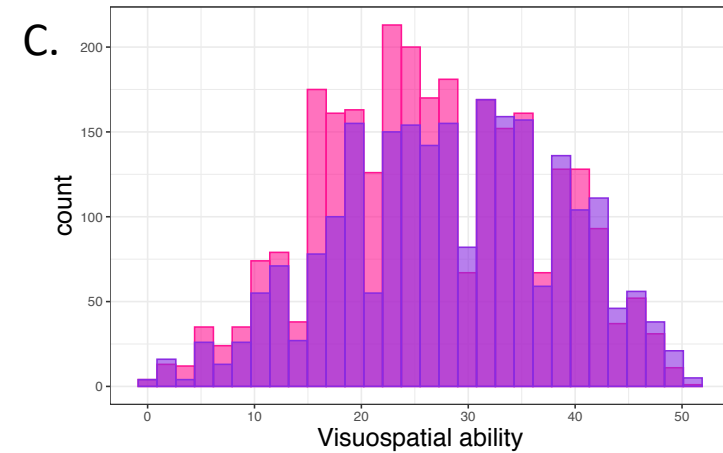

Sex

female

male

Supplement: Supplementary file 3 — Figure S2 [file 41398_2021_1649_MOESM3_ESM.pdf]
